# Health care barriers and perceived mental health among adults in Canada during the COVID-19 pandemic: a population-based cross-sectional study

**DOI:** 10.24095/hpcdp.44.1.03

**Published:** 2024-01

**Authors:** Mehrunnisa Shiraz, Colin A. Capaldi, Laura L. Ooi, Karen C. Roberts

**Affiliations:** 1 McGill University, Montral, Quebec, Canada; 2 Public Health Agency of Canada, Ottawa, Ontario, Canada

**Keywords:** access to health care, access to health services, health care seeking behaviour, health care utilization, mental health, COVID-19 pandemic, adults, Canada

## Abstract

**Introduction:**

The perceived mental health of individuals in Canada who faced health care barriers during the COVID-19 pandemic is underexplored.

**Methods:**

We analyzed data collected March to June 2021 from adults who reported needing health care services within the past 12 months in the Survey on Access to Health Care and Pharmaceuticals during the Pandemic. Unadjusted and adjusted logistic regression analyses examined the associations between health care barriers (appointment scheduling problems, delaying contacting health care) and high self-rated mental health and perceived worsening mental health compared to before the pandemic, overall and stratified by gender, age group, number of chronic health conditions and household income tertile.

**Results:**

Individuals who experienced pandemic-related appointment changes or had appointments not yet scheduled were less likely to have high self-rated mental health (aOR=0.81 and0.64, respectively) and more likely to have perceived worsening mental health (aOR=1.50 and 1.94, respectively) than those with no scheduling problems. Adults who delayed contacting health care for pandemic-related reasons (e.g. fear of infection) or other reasons were less likely to have high self-rated mental health (aOR=0.52 and0.45, respectively) and more likely to have perceived worsening mental health (aOR=2.31 and2.43, respectively) than those who did not delay. Delaying contacting health care for pandemic-related reasons was associated with less favourable perceived mental health in all subgroups, while the association between perceived mental health and pandemic-related appointment changes was significant in some groups.

**Conclusion:**

Health care barriers during the pandemic were associated with less favourable perceived mental health. These findings could inform health care resource allocation and public health messaging.

**Highlights**

- Adults in Canada who experienced health care appointment scheduling problems as a result of the COVID- 19 pandemic or whose appointments were not yet scheduled had less favourable perceived mental health than people who had no scheduling problems.
- Similarly, adults in Canada who delayed seeking health care during the pandemic had less favourable perceived mental health than those who did not delay seeking health care.
- Experiencing pandemic-related appointment changes (vs. no appointment scheduling problems) was associated with less favourable perceived mental health for older adults, individuals with chronic health conditions, and individuals from low or middle-income households, even after adjustment.
- Delaying contacting health care for pandemic-related reasons (vs. not delaying contact) was associated with less favourable perceived mental health across sociodemographic groups.

## Introduction

The COVID-19 pandemic brought about widespread changes to population mental health.^1^-^3^ Mental health is an umbrella term that encompasses a variety of clinical and subjective (or perceived) indicators and can include both positive mental health (defined as a state of well-being that allows us to “feel, think, and act in ways that enhance our ability to enjoy life and deal with the challenges we face”^4^^,p.1^) and mental illness.^5^ Studies have found that the prevalence of positive mental health outcomes (e.g. high self-rated mental health) declined and the prevalence of negative mental health outcomes (e.g. recent suicidal ideation) increased during the pandemic compared to 2019 in Canada.^1^-^3^ While numerous pandemic-related stressors may have influenced these trends, the aim of our study was to understand the link between health care barriers during the first year of the pandemic and perceived mental health.

Before the start of the pandemic, limitations in health care service availability—exemplified by long wait times and difficulties getting appointments—were among the most common health care accessibility challenges reported in Canada. 6-8 Data from the 2013 Canadian Community Health Survey indicate that 29% of individuals aged 15 years and older who received health care in the past year reported difficulties accessing these services.^7^

The response to the pandemic further complicated existing resource constraints, and resource allocation strategies prioritized urgent treatments.^9^ Other public health precautions, such as physical distancing, increased disinfection between patient appointments and pre-appointment COVID-19 screenings,^10^ also reduced health care system capacity. Preliminary findings from the Survey on Access to Health Care and Pharmaceuticals during the Pandemic (SAHCPDP) indicate that 49% of the adults in the Canadian provinces who reported needing physical and/or mental health care during the first year of the pandemic experienced difficulties accessing it.^11^ Specifically, 28% had at least one health care appointment cancelled, rescheduled, or delayed because of the pandemic, while 9% were unable to schedule at least one appointment.^11^

Along with service availability issues, 30% delayed contacting a medical professional, with fear of exposure to COVID-19 and concerns about overloading the health care system given as just two of the common reasons for such delays.^11^

Canada’s Quality of Life framework identifies health care barriers as detrimental to well-being by listing multiple indicators related to timely access to health care and unmet needs.^12^ In fact, findings from data collected in 1998/99 indicated that psychological distress in adults in Canada is associated with unmet health care needs due to barriers in health care availability, accessibility and acceptability.^6^ A study that used data from the 2002 Canadian Community Health Survey – Mental Health and Well-Being reported that perceived barriers to accessing mental health services were associated with lower self-reported coping ability and psychological well-being among individuals aged 15years and older who had recently experienced onset of a mental disorder.^13^

Surveys conducted during the pandemic in numerous countries suggest a link between difficulty accessing health care and experiencing negative mental health (e.g. symptoms of mental illness, such as anxiety and depression) among individuals with health conditions (i.e. individuals at high risk of severe illness from COVID-19 and persons with epilepsy).^14^,^15^ Moreover, data from June 2020 show that US adults who reported depression and anxiety symptoms had greater unmet health care needs due to the pandemic.^16^ However, a study of older adults in the Netherlands found that depression and anxiety symptoms were not significantly associated with appointment cancellations initiated by health care services.^17^

Studies have also explored the relationship between indicators of negative mental health and health care avoidance or delays in seeking health care during the pandemic. For instance, a study of US adults found that experiencing depression and anxiety symptoms was also associated with delaying medical care because of the pandemic.^16^ Furthermore, studies of older Dutch adults found that individuals who reported symptoms of depression or anxiety were more likely to delay contacting health care.^17^,^18^

Taken together, these findings broadly suggest a link between health care barriers and negative mental health indicators during the pandemic,^14^,^15^,^17^,^18^ particularly among subpopulations with greater health care needs (e.g. at high risk of severe illness from COVID-19, older adults, persons with epilepsy).^19^,^20^ However, there remain several gaps in our understanding of the associations between health care barriers and mental health among Canadians during the pandemic. Some studies used aggregate measures of health care barriers,^16^,^21^ but examination of specific types of health care barriers could provide results that are more actionable for decision makers. In addition, the existing research focusses on indicators of negative mental health; examining positive mental health and other perceived indicators of mental health (at the national level and among sociodemographic subpopulations) would offer a more complete understanding of Canadians’ mental health during the pandemic. Finally, most studies examined health care barriers relatively early in the pandemic and over a relatively short time,^14^-^16^,^18^ and therefore may have captured a smaller number of problems with health care accessibility and missed potential associations with mental health over a longer period.

The present study attempts to fill these evidence gaps by using data from the SAHCPDP to characterize the perceived mental health (i.e. self-rated mental health, perceived worsening mental health compared to before the pandemic) of adults in Canada who experienced different health care barriers (i.e. appointment scheduling problems, delayed contacting health care) during the first year of the pandemic. Given differences in mental health, health care accessibility and health care needs across sociodemographic factors,^6^,^7^,^19^,^20^,^22^ we also examined subpopulations that may be particularly affected by health care barriers. This study examined (1) the association between appointment scheduling problems and perceived mental health; (2) the association between delaying contacting a medical professional and perceived mental health; and (3) whether any such associations were found among different sociodemographic groups, including genders, age groups, chronic health condition status and household income levels.

## Methods

***Data source***

We used cross-sectional data from the SAHCPDP, collected from March to June 2021, with a target population of individuals aged 18 years and older residing in the Canadian provinces.^23^ The survey excluded institutionalized individuals and people living on reserves and other Indigenous settlements. The SAHCPDP obtained a main sample and an Indigenous oversample. For each, a simple random sample of dwellings was selected within each province and then an adult was selected from each dwelling. The main sample used the Dwelling Universe File as the sampling frame, whereas the oversample used a list of individuals who self-identified as Indigenous in the 2016 Census. The response rate was 46.2%, with a sample size of 25268.^23^ Of these respondents, 20620 agreed to share their data with the Public Health Agency of Canada (PHAC). Respondents completed the survey using either an online questionnaire or computer-assisted telephone interviewing.

Because this study was based on aggregated, deidentified secondary data shared with PHAC under the purview of the federal *Statistics Act*, ethics approval was not required.

***Eligibility criteria***

We restricted analyses to those who reported needing one or more physical and/or mental health care services during the past 12 months (n = 17335). We removed proxy interviews to ensure that perceived mental health indicators were self-reported, resulting in a sample size of 17199.

***Measures***

**High self-rated mental health**

Self-rated mental health was measured with the question “In general, how is your mental health?” Respondents could select one of the following: “Excellent,” “Very good,” “Good,” “Fair” or “Poor.” Those who selected “Excellent” or “Very good” were coded as having high self-rated mental health.^24^,^25^

**Perceived worsening mental health**

Perceived worsening mental health was assessed with the question “Compared to before the COVID-19 pandemic, how would you say your mental health is now?” Respondents could select one of the following: “Much better now,” “Somewhat better now,” “About the same,” “Somewhat worse now” or “Much worse now.” Individuals who selected “Somewhat worse now” or “Much worse now” were coded as having perceived worsening mental health.^26^,^27^

**Appointment scheduling problems**

Respondents were asked “Did you experience any of the following problems with the scheduling of your appointments?” Response options were “One or more of your appointments was cancelled, rescheduled or delayed due to the COVID-19 pandemic,” “One or more of your appointments was cancelled, rescheduled or delayed due to other reasons,” “One or more of your appointments has not been scheduled yet” and “Did not experience any problems with the scheduling of your appointments.” Respondents could select more than one type of appointment scheduling problem.

In the overall analyses, we categorized respondents into one of four groups: (1)no appointment scheduling problems (reference group); (2) at least one pandemic-related appointment change (referred to as “pandemic-related appointment changes”); (3) at least one appointment change, but unrelated to the pandemic (referred to as “non-pandemic appointment changes”); and (4) at least one appointment that had not yet been scheduled, but no appointment changes (referred to as “appointments not yet scheduled”).

In the stratified analyses, we only examined individuals who experienced pandemic-related appointment changes and individuals who had no appointment scheduling problems. We excluded the remaining groups due to insufficient sample sizes.

**Delays in contacting health care**

Respondents were asked “In the past 12 months, did you delay contacting a medical professional about a problem with your physical, emotional or mental health for any of the following reasons?” Response options were “Fear of possible COVID-19 exposure in health care settings,” “Fear of possible COVID-19 exposure outside of health care settings,” “Concern of overloading the health care system,” “Other” and “Did not delay contacting a medical professional.” Respondents could select multiple reasons for delaying contact.

In the overall analyses, we categorized respondents into one of three groups: (1)did not delay contacting a medical professional (reference group); (2) delayed contacting a medical professional at least in part for pandemic-related reasons; and (3)delayed contacting a medical professional for other reasons only. In the stratified analyses, we did not include the group of respondents who delayed contacting a medical professional for other reasons only because of insufficient sample sizes.

**Covariates**

Several variables can confound the relationship between health care barriers and perceived mental health.^3^,^6^,^7^,^11^,^19^,^20^,^22^,^28^-^32^ We statistically controlled for gender (man, woman; the gender-diverse category was excluded due to insufficient sample sizes), age (continuous), ethnicity (White, Indigenous, non-Indigenous racialized group member), immigrant status (born in Canada, born outside Canada), number of diagnosed chronic health conditions (0, 1, or 2+ of the 16, including “Other,” listed in the SAHCPDP questionnaire^33^), household income tertile (low [<$60 000], middle [$60000–$110000], high [≥ $110 000], derived from respondents’ estimates of their total household income before taxes in the previous year33) and geographic location (British Columbia, the Prairie provinces [Alberta, Saskatchewan and Manitoba], Ontario, Quebec, Atlantic provinces [New Brunswick, Prince Edward Island, Nova Scotia, Newfoundland and Labrador]). 

**Analyses**

All analyses were conducted using statistical package SAS Enterprise Guide version 7.1 (SAS Institute, Cary, NC, US). To account for the survey design and ensure the findings were representative of the target population, we used sampling weights. We estimated 95% confidence intervals, standard errors and coefficients of variation using bootstrap weights. Statistics Canada provided the sampling and bootstrap weights.

We calculated the overall prevalence of the different categories of appointment scheduling problems, delays in contacting health care, high self-rated mental health, perceived worsening mental health and the covariates. We also computed the prevalence of high self-rated mental health, perceived worsening mental health, categories of appointment scheduling problems and delays in contacting health care by gender, age group (young adults [18–34 years], middle-aged adults [35–64 years] and older adults [65+ years]), number of chronic health conditions and household income tertile. We also computed the prevalence of high self-rated mental health and perceived worsening mental health by each category of appointment scheduling problems and delays in contacting health care, overall and stratified by sociodemographic groups. We interpreted prevalence estimates with non-overlapping confidence intervals as significantly different.

We fitted unadjusted and adjusted logistic regression models to examine the associations between (1) appointment scheduling problems and high self-rated mental health; (2) appointment scheduling problems and perceived worsening mental health; (3) delaying contacting health care and high self-rated mental health; and (4)delaying contacting health care and perceived worsening mental health. Each unadjusted model included only the main explanatory variable of interest, whereas adjusted models included the main explanatory variable and the covariates. To examine these associations in subpopulations, the unadjusted and adjusted logistic regression analyses were also stratified by gender, age group, number of chronic health conditions and household income tertile. We interpreted odds ratios with 95% confidence intervals that do not include 1.00 as statistically significant. When comparing odds ratios across stratified regression models, we interpreted odds ratios with non-overlapping confidence intervals as significantly different. The overall and stratified regression analyses (and their corresponding prevalence estimates) were restricted to complete case records for the relevant variables. Each pair of unadjusted and adjusted regression models were based on the same respondents.

## Results

***Descriptive statistics***

Table 1 shows the distribution of sociodemographic characteristics in the study population.

**Table 1:** Sociodemographic characteristics of individuals who reported needing health care services during the past 12 months, March 2021–June 2021, Canada

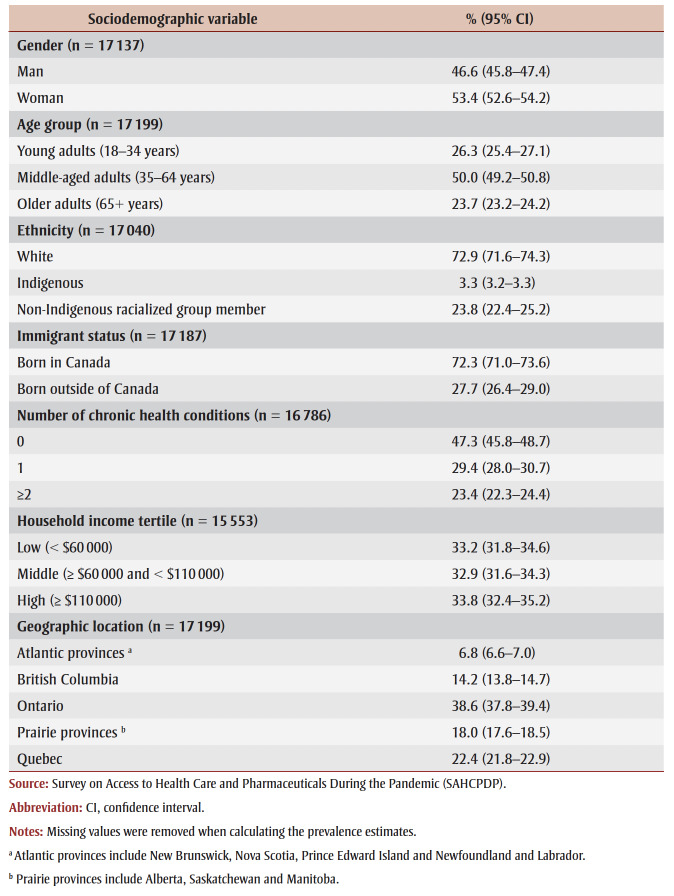

The overall and stratified prevalence of the health care barriers and perceived mental health indicators are shown in Table 2. Overall, 42.9% (95% CI: 41.5–44.3) of adults who reported needing a health care service in the past 12 months had high self-rated mental health, with lower proportions of women (39.3%; 37.6–41.1) than of men (47.2%; 44.9–49.5) and adults with one (39.1%; 36.6–41.7) and multiple (34.6%; 32.2–37.0) chronic conditions than with no chronic conditions (49.4%; 47.1–51.8) reporting high self-rated mental health. The prevalence of high self-rated mental health was highest among older adults (52.2%; 50.0–54.5) and lowest among young adults (34.5%; 31.1–37.8). 

**Table 2:** Prevalence of perceived mental health indicators, appointment scheduling problems and delays in contacting health care, overall and stratified by gender, age group, number of chronic health conditions and household income tertile, Canada

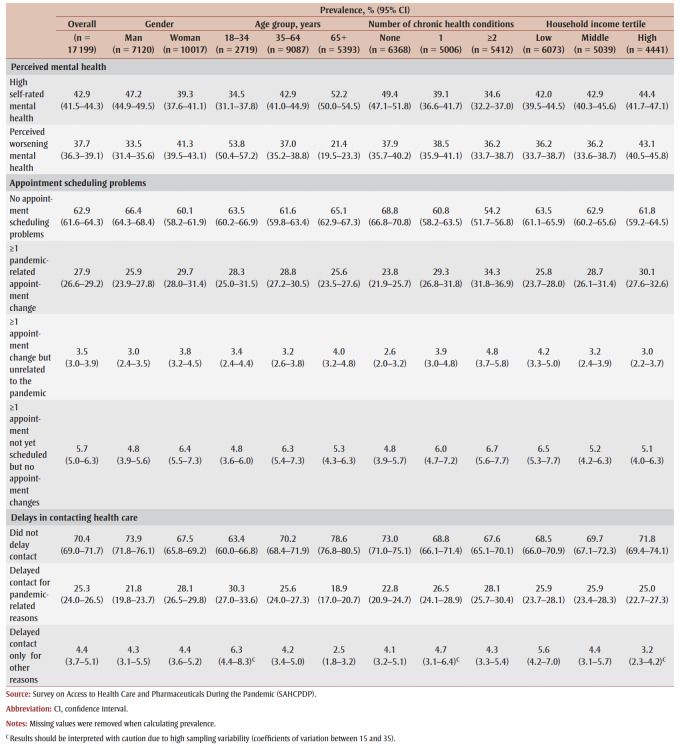

Moreover, 37.7% (95% CI: 36.3–39.1) of adults who reported needing health care services also reported perceived worsening mental health, with higher proportions of women (41.3%; 39.5–43.1) than of men (33.5%; 31.4–35.6) and people in the high household income tertile (43.1%; 40.5–45.8) than the low (36.2%; 33.7–38.7) and middle (36.2%; 33.6–38.7) household income tertiles reporting perceived worsening mental health. The prevalence of perceived worsening mental health was highest among young adults (53.8%; 50.4–57.2) and lowest among older adults (21.4%; 19.5–23.3). Further, 27.9% (26.6–29.2) experienced at least one pandemic-related appointment change, with higher proportions of women (29.7%; 28.0–31.4) than of men (25.9%; 23.9–27.8), and those with one (29.3%; 26.8–31.8) and multiple (34.3%; 31.8–36.9) chronic conditions versus no chronic conditions (23.8%; 21.9–25.7) reporting one or more pandemic-related appointment changes. 

Overall, 25.3% (95% CI: 24.0–26.5) of respondents delayed contacting health care for pandemic-related reasons. Higher proportions of women (28.1%; 26.5–29.8) than of men (21.8%; 19.8–23.7), young (30.3%; 27.0–33.6) and middle-aged (25.6%; 24.0–27.3) than of older adults (18.9%; 17.0–20.7) and adults with multiple chronic conditions (28.1%; 25.7–30.4) versus no chronic conditions (22.8%; 20.9–24.7) delayed contacting health care for pandemic-related reasons.

***Appointment scheduling problems and perceived mental health***

The regression results for appointment scheduling problems are presented in Table 3.

**Table 3:** Unadjusted and adjusted associations between appointment scheduling problems and perceived mental health indicators, overall and stratified, Canada

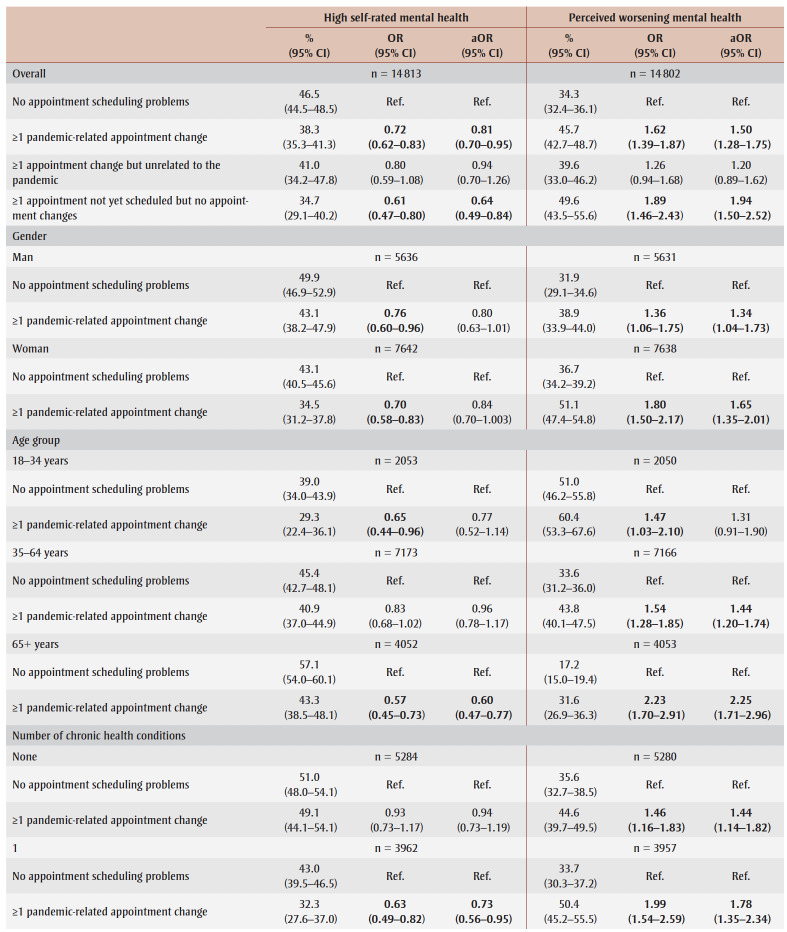 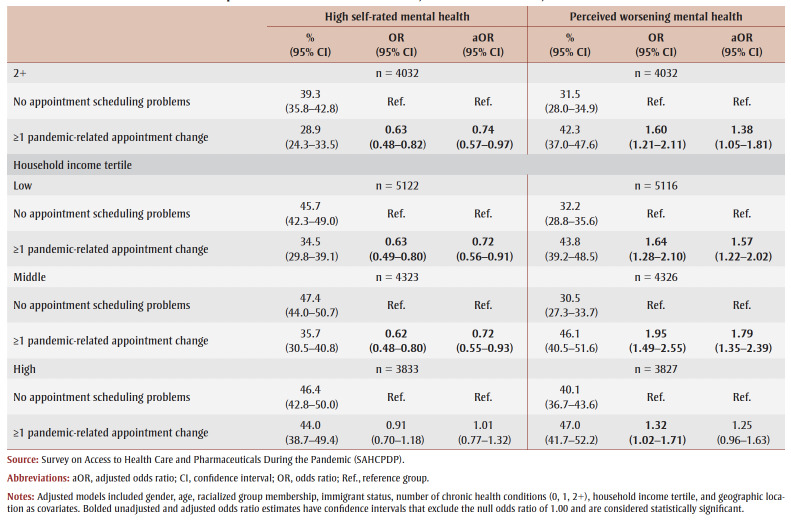

Overall, compared to those who experienced no appointment scheduling problems, those who faced pandemic-related appointment changes (OR = 0.72, 95% CI: 0.62–0.83; aOR = 0.81, 95% CI: 0.70–0.95) and those with appointments not yet scheduled (OR = 0.61, 0.47–0.80; aOR = 0.64, 0.49–0.84) had lower odds of reporting high self-rated mental health before and after adjusting for covariates. The odds of high self-rated mental health did not differ significantly between those who experienced non-pandemic appointment changes and those who experienced no appointment scheduling problems (Table 3). 

Stratified analyses indicated that, compared to those who experienced no appointment scheduling problems, the unadjusted odds of reporting high self-rated mental health were significantly lower for those who experienced pandemic-related appointment changes among men (OR = 0.76, 95% CI: 0.60–0.96), women (OR = 0.70, 0.58–0.83) and younger adults (OR = 0.65, 0.44–0.96) (Table 3). 

Both the unadjusted and adjusted odds of reporting high self-rated mental health were significantly lower for those who experienced pandemic-related appointment changes among older adults (OR = 0.57, 95% CI: 0.45–0.73; aOR = 0.60, 95% CI: 0.47–0.77), those with 1 chronic condition (OR = 0.63, 0.49–0.82; aOR = 0.73, 0.56–0.95) and 2+ chronic conditions (OR = 0.63, 0.48–0.82; aOR = 0.74, 0.57–0.97), and those in low-income households (OR=0.63, 0.49–0.80; aOR = 0.72, 0.56–0.91) and middle-income households (OR = 0.62, 0.48–0.80; aOR = 0.72, 0.55–0.93). The odds of high self-rated mental health did not differ between the two appointment scheduling groups for middle-aged adults, those with no chronic conditions and those in high-income households (Table 3).

Overall, compared to those who had no appointment scheduling problems, respondents who experienced pandemic-related appointment changes (aOR = 1.50, 95% CI: 1.28–1.75) and those with appointments that were not yet scheduled (aOR = 1.94, 1.50–2.52) had greater odds of reporting perceived worsening mental health, before and after adjusting for covariates. However, the odds of perceived worsening mental health did not significantly differ between those who faced non-pandemic appointment changes and those who did not experience any appointment scheduling problems (Table 3). 

The unadjusted and adjusted odds of reporting perceived worsening mental health were significantly greater among those who faced pandemic-related appointment changes compared to those who faced no appointment scheduling problems among men (OR = 1.36, 95% CI: 1.06–1.75; aOR= 1.34, 95% CI: 1.04–1.73), women (OR = 1.80, 1.50–2.17; aOR = 1.65, 1.35–2.01), those with no chronic conditions (OR = 1.46, 1.16–1.83; aOR = 1.44, 1.14–1.82), 1 chronic condition (OR = 1.99, 1.54–2.59; aOR = 1.78, 1.35–2.34), and 2+ chronic conditions (OR = 1.60, 1.21–2.11; aOR = 1.38, 1.05–1.81), those in low-income households (OR = 1.64, 1.28–2.10; aOR = 1.57, 1.22–2.02) and middle-income households (OR = 1.95, 1.49–2.55; aOR= 1.79, 1.35–2.39), and middle-aged (OR = 1.54, 1.28–1.85; aOR = 1.44, 1.20–1.74) and older adults (OR = 2.23, 1.70–2.91; aOR = 2.25, 1.71–2.96). The unadjusted odds of reporting perceived worsening mental health were also significantly higher for young adults (OR=1.47, 1.03–2.10) and those in high-income households (OR= 1.32, 1.02–1.71) who experienced pandemic-related appointment changes (Table 3).

***Delays in contacting health care and perceived mental health***

The regression results for delays in contacting health care are presented in Table 4.

**Table 4:** Unadjusted and adjusted associations between delaying contacting health care and perceived mental health indicators, overall and stratified, Canada

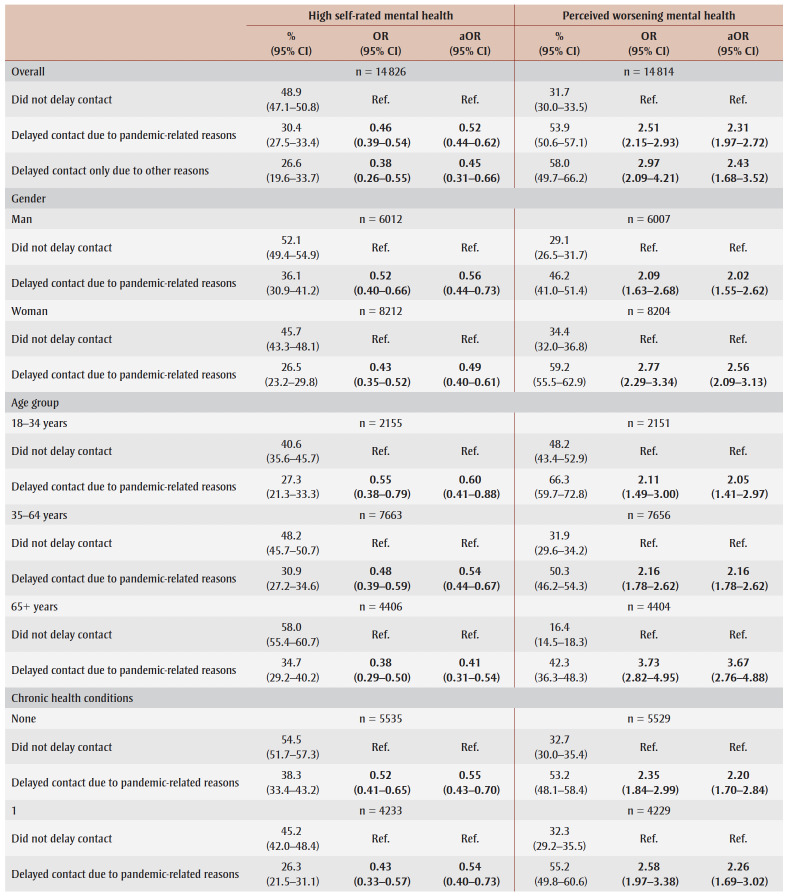 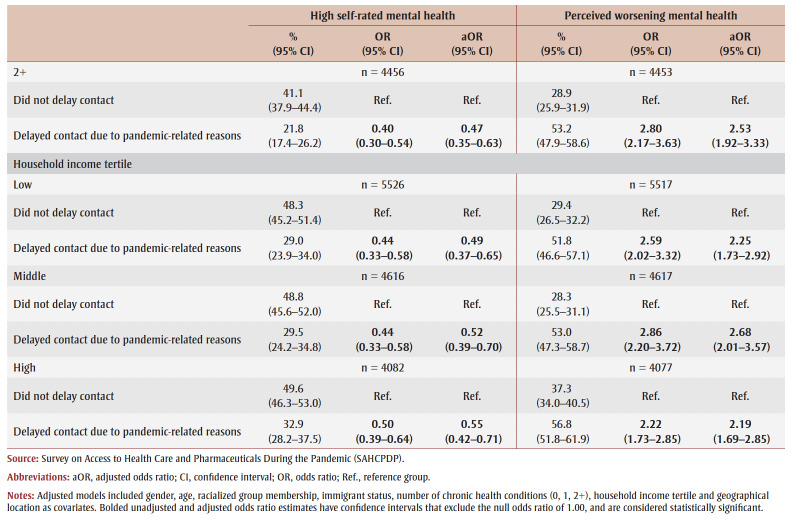

Overall, compared to those who did not delay contacting health care, those who delayed contacting health care for pandemic-related reasons (OR = 0.46, 95% CI: 0.39–0.54; aOR = 0.52, 95% CI: 0.44–0.62) and those who delayed only for other reasons (OR = 0.38, 0.26–0.55; aOR = 0.45, 0.31–0.66) were less likely to report high self-rated mental health, before and after adjustment (Table 4). 

The unadjusted and adjusted odds of high self-rated mental health were significantly lower among those who delayed contacting health care for pandemic-related reasons compared to those who did not delay contacting health care (OR range from 0.38–0.55; aOR range from 0.41–0.60) across gender, age group, household income tertile and chronic health condition categories (Table 4).

Overall, compared to those who did not delay contacting health care, the odds of reporting perceived worsening mental health were significantly greater among those who delayed contacting health care for pandemic-related reasons (OR = 2.51, 95% CI: 2.15–2.93; aOR = 2.31, 95% CI: 1.97–2.72) and for other reasons (OR = 2.97, 2.09–4.21; aOR = 2.43, 1.68–3.52), before and after adjustment. The unadjusted and adjusted odds of perceived worsening mental health were significantly greater among those who delayed contacting health care for pandemic-related reasons compared to those who did not delay contacting health care (OR range from 2.09–3.73; aOR range from 2.02–3.67) across gender, age group, household income tertile and chronic health condition categories. Notably, the association between delaying contact due to pandemic-related reasons and perceived worsening mental health was stronger among older adults (OR = 3.73, 2.82–4.95; aOR = 3.67, 2.76–4.88) than among middle-aged adults (OR = 2.16, 1.78–2.62; aOR = 2.16, 1.78–2.62) (Table 4).

## Discussion

The COVID-19 pandemic led to reduced health care provision for non-COVID-19 health issues,^9^ and decreased willingness among some to seek health care because of concerns about contracting COVID-19 or overloading the health care system.^11^ These widespread changes in health care availability and health care–seeking behaviours during the pandemic coincided with declines in population mental health.^1^-^3^ Our study examined whether experiencing health care barriers might be one explanation for declines in perceived mental health. We found that several health care barriers were associated with less favourable perceived mental health indicators; these included pandemic-related appointment changes, having appointments not yet scheduled, and delaying contact with health care for pandemic-related or other reasons.

Beyond preventing mortality and disease, timely access to health care is a key resource for achieving mental and physical well-being.^12^ The vast majority of those who experienced difficulties accessing health care during the pandemic reported that this had a negative impact on their life, with worry, stress or anxiety; pain; worsening health and problems with daily living activities being commonly reported impacts.^11^ Experiencing appointment scheduling problems could negatively affect perceived mental health directly and/or indirectly by influencing other determinants of mental health (e.g. health status, physical activity, coping ability, sense of control and self-efficacy).^25^ Future research could explore how prolonged health care wait times might be associated with additional indicators of mental health (and their determinants), including positive mental health outcomes beyond high self-rated mental health and/or validated measures of mental illness.

Recognizing that the impacts of health care barriers can vary across subgroups, we stratified our analyses by gender, age group, number of chronic health conditions and household income tertile. We found that the relationship between pandemic-related appointment changes and both indicators of perceived mental health persisted for older adults, individuals with one or multiple chronic health conditions, and those in low- and middle-income households. Since these subpopulations tend to have greater health care needs,^19^,^34^ pandemic-related appointment changes might have had a disproportionate impact on their perceived mental health.^12^ These findings can inform decision makers about health care resource allocation by focussing on the potential mental health consequences of prolonged wait times, especially for those with the greatest needs.

Deciding to postpone addressing one’s current health care needs to avoid risking COVID-19 exposure or overloading the health care system may cause individuals to worry and feel stressed. Our results show that adults in Canada who delayed contacting health care for pandemic-related or other reasons were less likely to report high self-rated mental health and more likely to report perceived worsening mental health than those who did not delay contacting health care. Moreover, across all sociodemographic groups, those who delayed contacting health care for pandemic-related reasons had less favourable perceived mental health compared to those who did not delay. 

These findings add to the existing literature showing that greater COVID-19 risk perception is associated with worse mental health, including a lower likelihood of reporting high self-rated mental health,^35^ and increased depression and anxiety symptoms.^36^ The uncertainty and stress of having unmet health care needs and deteriorating health resulting from delaying health care may compound these effects. Indeed, recent research found that older US adults with low self-rated mental health or low self-rated physical health were more likely to perceive negative health effects due to delayed health care.^21^ Delaying contacting health care could also reflect an avoidance coping style, which has been associated with higher levels of depression and anxiety symptoms during the pandemic.^37^

The robust association between delaying contacting health care and less favourable perceived mental health in our study highlights the importance of continued public health messaging encouraging people to seek health care that they need. Primary care providers and other clinicians may play an important role in counselling patients who are hesitant to seek care and in addressing COVID-19 safety concerns. Access to alternative care modalities, like telehealth,^38^ as well as implementing health and safety measures, such as COVID-19 symptom screening, and informing potential patients of these might reduce the prevalence of delays in contacting health care. Additional research could explore other reasons for delaying health care during the pandemic and identify points for intervention. Future Canadian research might also consider other subpopulations experiencing barriers to seeking health care during the pandemic, such as those identified in a US study (unpaid caregivers for adults, people with disabilities and racialized group members).^39^

***Strengths and limitations***

Our study provides a nuanced examination of the relationship between various types of health care barriers and different indicators of perceived mental health among adults in Canada during the pandemic. The large, population-based sample allowed us to examine these associations across different sociodemographic groups and control for important covariates.

This study is not without limitations. The SAHCPDP used a cross-sectional design, which precludes inferring the directionality and causality of findings. The response rate for the SAHCPDP was under 50%, but survey weights were developed to mitigate biases associated with nonresponse. Children and youth, institutionalized populations and people living in the territories, on reserves or in other Indigenous settlements were not included in the survey, which affects the generalizability of the findings; these population groups may also be vulnerable to the effects of health care barriers.^40^-^42^

The survey was self-reported, and therefore susceptible to misclassification of household income and recall bias with respect to health care experiences and needs. The reported household income may not reflect respondents’ typical, pre-pandemic income if they experienced employment loss or were able to make use of the COVID-19 relief programs available during 2020.^43^ In addition, some stratified analyses had covariates with high sampling variability. 

We were only able to examine one positive mental health indicator, which limits a broader understanding of the relationship between health care barriers and positive mental health (and overall mental health). Our study aggregated health care barriers across all health care services, but the mental health implications of health care barriers may differ depending on the type of service (e.g. urgent vs. routine, physical vs. mental). It is also unclear how many appointment changes were initiated by the health care provider or the patient. The way the appointment was changed—whether it was cancelled, rescheduled or delayed—is also unknown. We aggregated the experiences of individuals who delayed contacting health care due to concerns about COVID-19 infection and due to concerns about burdening the health care system. These varied pandemic-related motivations could have differing implications for different facets of mental health. Finally, we applied a highly conservative approach to testing differences among prevalences and ORs by using non-overlapping CIs to highlight statistically significant differences of interest.

## Conclusion

During the first year of the COVID-19 pandemic, several health care barriers, including pandemic-related appointment changes, appointments not yet scheduled and delaying contacting health care for any reason were negatively associated with high self-rated mental health and positively associated with perceived worsening mental health. The association between pandemic-related appointment changes and unfavourable perceived mental health persisted across sociodemographic groups that tend to have greater health care needs. On the other hand, the relationship between delaying contacting health care for pandemic-related reasons and unfavourable perceived mental health persisted for all examined sociodemographic groups. As we navigate the recovery period of the pandemic, continued surveillance is necessary to track the prevalence of health care barriers and mental health in the Canadian population.

## Acknowledgements

The authors would like to thank Mlanie Varin for her help in finalizing the manuscript.

## Conflicts of interest

The authors have no conflicts of interest.

## Authors’ contributions and statement

MS – Methodology, formal analysis, writing – original draft, writing – review & editing.

CC – Conceptualization, methodology, validation, writing – review & editing, supervision.

LO – Methodology, validation, writing – review & editing, supervision.

KCR – Methodology, writing – review & editing, supervision.

The content and views expressed in this article are those of the authors and do not necessarily reflect those of the Government of Canada.
